# Rescue of NBD2 Mutants N1303K and S1235R of CFTR by Small-Molecule Correctors and Transcomplementation

**DOI:** 10.1371/journal.pone.0119796

**Published:** 2015-03-23

**Authors:** Daniele Rapino, Inna Sabirzhanova, Miquéias Lopes-Pacheco, Rahul Grover, William B. Guggino, Liudmila Cebotaru

**Affiliations:** 1 Department of Medicine, Johns Hopkins University, School of Medicine, Baltimore, Maryland, United States of America; 2 Department of Physiology, Johns Hopkins University, School of Medicine, Baltimore, Maryland, United States of America; Odense University hospital, DENMARK

## Abstract

Although, the most common Cystic Fibrosis mutation, ΔF508, in the cystic fibrosis transmembrane regulator. (CFTR), is located in nucleotide binding domain (NBD1), disease-causing mutations also occur in NBD2. To provide information on potential therapeutic strategies for mutations in NBD2, we studied, using a combination of biochemical approaches and newly created cell lines, two disease-causing NBD2 mutants, N1303K and S1235R. Surprisingly, neither was rescued by low temperature. Inhibition of proteasomes with MG132 or aggresomes with tubacin rescued the immature B and mature C bands of N1303K and S1235R, indicating that degradation occurs via proteasomes and aggresomes. We found no effect of the lysosome inhibitor E64. Thus, our results show that these NBD2 mutants are processing mutants with unique characteristics. Several known correctors developed to rescue ΔF508-CFTR, when applied either alone or in combination, significantly increased the maturation of bands B and C of both NBD 2 mutants. The best correction occurred with the combinations of C4 plus C18 or C3 plus C4. Co-transfection of truncated CFTR (∆27-264) into stably transfected cells was also able to rescue them. This demonstrates for the first time that transcomplementation with a truncated version of CFTR can rescue NBD2 mutants. Our results show that the N1303K mutation has a more profound effect on NBD2 processing than S1235R and that small-molecule correctors increase the maturation of bands B and C in NBD2 mutants. In addition, ∆27-264 was able to transcomplement both NDB2 mutants. We conclude that differences and similarities occur in the impact of mutations on NBD2 when compared to ΔF508-CFTR suggesting that individualized strategies may be needed to restore their function. Finally our results are important because they suggest that gene or corrector molecule therapies either alone or in combination individualized for NBD2 mutants may be beneficial for patients bearing N1303K or S1235R mutations.

## Introduction

Cystic Fibrosis is a recessive autosomal disorder caused by mutations in the cystic fibrosis transmembrane conductance regulator (CFTR), an ATP-binding cassette protein (ABC, sub-family C, member 7) composed of two transmembrane domains, two nucleotide-binding domains, and a unique regulatory domain. CFTR functions as a chloride channel in epithelial cells where, depending on the tissue, transports chloride ions either into or out of the cells [1]. This function is very important for controlling the water balance in the mucosal membranes of the airways the digestive and reproductive systems. The most common clinical manifestations of cystic fibrosis are recurrent pulmonary infection, pancreatic insufficiency, diabetes mellitus, and male infertility [2].

Numerous disease-causing mutations (over 1000) have been described; the most common is a deletion of phenylalanine at position 508 (ΔF508) in the NBD1 domain, [3] which results in severe CF. This amino acid deletion diminishes both the thermal stability of NBD1 and its ability to interact properly with the transmembrane domains [4]. ΔF508-CFTR is retained in the ER, and thus it is only core-glycosylated [5]. It is degraded rapidly in the proteasome [6]. Recently, the FDA has approved Kalydeco (VX-770) for patients with the G551D mutation, which is also associated with severe CF [7]. Unlike ΔF508-CFTR, G551D is a gating mutant that traffics to the plasma membrane but requires potentiation of its defective channel activity [8]. Patients taking Kalydeco, which was derived from a basic understanding of the need of potentiation for the G551D mutant channel function [9], have shown remarkable improvement in most of the clinical manifestations of their disease [7]. However, correction of ΔF508-CFTR trafficking has proved more difficult [10]. For example, VX-809, a small molecule developed to rescue ΔF508-CFTR processing and trafficking, has been used in a Phase IIa clinical trial in ΔF508-homozygous patients but has demonstrated only limited clinical efficacy [11]. Thus, either a new class of correctors will have to be identified, or VX-809 will have to be combined with other small molecules to reach therapeutic level.

There has been much interest in the gating and trafficking mutations in NBD1, such as G551D and ΔF508-CFTR. We have recently shown that another NBD1 mutant, A455E, can be rescued [12]. However, the question arises as to whether therapies developed for mutations in NBD1 will be effective for use with mutations in NBD2. NBD1 and 2 form an intimate interaction that is crucial for ATP binding and hydrolysis, thereby ultimately regulating channel gating [13]. Mutations in NBD1 are often also manifested by changes in NBD2 [14,15]. However, NBD2 is translated and reaches its mature conformation much later than NBD1 [15,16]. Because of this temporal difference, it is unclear whether strategies developed to rescue mutations in NBD1 will also rescue NBD2. To address this question, we have now focused on two NBD2 mutations, N1303K and S1235R.

N1303K has a high incidence in Europe, and it has been reported to be the second most common mutation in Italy (4%). In some regions the incidence of this mutation is even higher, for example, reaching 7.8% in the southwestern region of France [17]. N1303K is classified as a severe mutation with respect to the pancreas (causing pancreatic insufficiency and diabetes mellitus) [18]; it is also associated with precocious and severe lung symptoms such as newborn pneumothorax [19]. The incidence of S1235R is approximately 1% (3.3% in Eastern Europe), significantly higher than other rare mutations. Phenotypic manifestations appear to be milder and more variable than those associated with N1303K and are mainly related to chronic pancreatitis, associated with sweat chloride test results [20–23] that are on the borderline of what is considered to be typical of CF. Taken together, these two mutations account for a significant percentage of CF mutations other than ΔF508.

We have now tested two strategies to rescue NBD2 mutants, small-molecule correctors or transcomplementation. In addition to VX-809, there are several correctors that have been identified and are available through the CF Foundation Therapeutics (CFFT). In this study, we have studied C3 and C18 (both identified by Vertex) [24] and C4 (identified by Pedimonte and his collaborators) [25]; as well as CFFT-003 and 004 [26]. In addition, our group has been developing a novel gene therapeutic approach for correcting CFTR processing mutants, based on transcomplementation, in which truncated forms of CFTR coexpressed with ΔF508-CFTR are able to rescue it by forming a bimolecular complex or by displacing chaperones from the mutant ΔF508-CFTR. This strategy allows CFTR to traffic to the plasma membrane restoring its function [27]. Our data show that small-molecule correctors can rescue NBD2 mutants, but they function better when used in combination rather than individually. In addition we demonstrate that transcomplementation approach can also rescue NBD2 mutants.

## Methods

### Cell culture

The African green monkey kidney fibroblast-like cell line (COS-7) was maintained in Dulbecco’s modified Eagle’s medium (DMEM) with penicillin (100 U/ml), streptomycin (100 μg/ml), and 10% fetal bovine serum. Human embryonic kidney (HEK) 293 cells (Catalog#CRL-1573, Life Technologies) were maintained in DMEM High Glucose 1x with penicillin (100 U/ml), streptomycin (100 μg/ml), hygromycin B (100 μg/ml) and 10% fetal bovine serum. The human embryonic kidney Flp-In-293 cell line was used to generate two stable cell lines expressing the PBI CMV_2_ N1303K CFTR and PBI CMV_2_ S1235R CFTR plasmids (provided by Phil Thomas (U. Texas, Southwestern). The use of stable cell lines resulted in uniformity of expression of the mutants among individual samples in each experiment.

### Plasmids and transfection

The mutants were studied either by transient transfection into Cos-7 cells or by stable expression using the HEK Flp-In-293 cells. PBI CMV_2_ N1303K CFTR, pc-DNA Δ27–264, PBI CMV_2_ S1235R CFTR, PBI CMV_2_ CFTR and PBI CMV_2_ ΔF508 plasmids were used to perform transient transfections using Lipofectamine 2000. The transfection was performed according to the Invitrogen protocol. Cells were grown in the regular DMEM medium to be 70–90% confluent at the transfection. Lipofectamine 2000 was diluted in the Opti-MEM medium. DNA was diluted in the DMEM medium. After 5 minutes of incubation at room temperature, the eluted lipofectamine was mixed with the eluted DNA. This solution was incubated for 20 minutes at room temperature. During this time the medium used to culture the cells was replaced with the Opti-MEM medium. After the 20 minutes incubation the solution with DNA and lipofectamine was poured in the 6-well dishes and incubated at 37°C. After 5 hours the Opti MEM medium with the diluted Lipofectamine and DNA was replaced with the regular DMEM medium. 48 hours after transfection, cells were harvested and used for western blotting.

### Western blotting

The cells were harvested using lysis buffer (50 mM NaCl, 150 mM Tris-HCl, pH 7.4, and 1% Nonidet P-40) with protease inhibitors (Roche Applied Science). Lysates were centrifuged for 20 min at 15000 rpm. The pellets were discarded, and the supernatants were collected and used to perform western blotting. CFTR was detected with monoclonal mouse IgG1 217directed against the R domain (aa 807–819), diluted 1:1000, or monoclonal mouse IgG2b 596 (217 & 596 obtained from Jack Riordan at the U. of North Carolina at Chapel Hill) against the NBD2 (aa 1204–1211), diluted 1:1000. Ezrin protein was used as the loading control detected by monoclonal mouse antibody (Santa Cruz). As secondary antibody, monoclonal mouse IgG1 (diluted 1:10000; Santa Cruz) was used. The signal was enhanced with Pierce ECL Western Blotting Substrate. Chemoluminescence was captured by a Fuji Film LAS-4000 plus system with a cooled CCD camera. Quantification was carried out within the linear range using Image Gauge version 3.2 software (Fuji Film).

### Co-Immunoprecipitation

After protein extraction as described above, co-immunoprecipitation was performed: 80 μl of protein A/G-agarose beads (SantaCruz Biotechnology) were prewashed with lysis buffer plus protease inhibitor four times. Cell lysates (2000 μg) were rotated with anti-CFTR antibody M3A7 (Millipore) and A/G beads for 4 h at 4°C. The samples were centrifuged at 2000 rpm for 2 min on a high speed table top centrifuge, the supernatants were discarded, and the A/G beads were washed with lysis buffer plus protease inhibitor four times. Sample buffer 2X plus β-mercaptoethanol was added 1:1 with the beads. Samples were used for western blotting as described above using the appropriate antibodies for each individual protein co-immunoprecipitated. CFTR precipitation (pull-down with antibody M3A7 and blot with 596) was used as a control. Rabbit polyclonal antibodies were used for VCP and HDAC6 (1:200); mouse monoclonal for HSP27 (1:2000), HSP40 and 70 (1:1000) and HSP90 (1:500) (Santa Cruz Bioltechnology)

### Biotinylation

Surface proteins were labeled with biotin (Thermo Scientific) for 20 min at 4°C, then washed with Glycine 200mM for 3 times. After cells were lysed, 1500 μg of proteins were incubated with avidin beads (NeutrAvidin Ultra Link Resin, Thermo Scientific) for 1 h at 4°C to isolate surface-labeled proteins. The samples were washed with lysis buffer, 4 times. Supernatants were discarded, and Laemmli sample buffer 2X plus β-mercaptoethanol was added 1:1 to the avidin beads. Samples were used to perform western blotting. As an internal control, ezrin detected with a monoclonal mouse antibody was used.

### Small-molecule correctors and inhibitors

We tested compounds C3, C4, and C18, obtained from the CFFT panel library (www.cftrfolding.org), as well as CFFT-002 and CFFT-003, obtained from Martin Mense (CFFT). In addition, we used compounds C3, C4, and C18 in combination of two. The effect of proteasome, aggresome, and lysosome inhibitors (MG132, tubacin, and E64, respectively) on the N1303K and S1235R mutations was also studied. Initially, a dose-response experiment with incremental doses was performed: 1, 5, 10, and 20 μM for the three correctors and tubacin and 1, 10, 50, and 100 μM for E64. The best two doses were repeated at least three times.

### Short-circuit current measurements

Short-circuit current (I_SC_) measurements were conducted using a six-channel Easy-Mount chamber system (Physiologic Instruments, San Diego, CA). CFBE410- cells (a gift of Dr. Dieter Gruenert, University of California, San Francisco) were stably transfected with N1303K using Flp-In technology. Once the cell line was established and verified to contain N1303K by western blotting, the cells were grown on Snapwell filters (Corning Costar, Acton, MA; 3407). The cells were infected with either 10 μl of AAV1 containing Δ264-CFTR (8–9 X 10^12^ vector genomes/ml, produced by the CF Vector Core at the U. of Florida using standard techniques [28]) for 3 days, and Isc was measured 1 week after infection. I_SC_ was measured with a VCCMC6 multichannel voltage-current clamp amplifier (Physiologic Instruments). Data were acquired on an 1.71-GHz PC running Windows XP (Microsoft, Redmond, WA) and equipped with DI-720 (DATAQ Instruments, Akron, OH), with Acquire and Analyze version 2.3.159 (Physiologic Instruments) software. The cell monolayers were bathed on both sides with solution containing 120 mM NaCl, 5 mM KCl, 2 mM MgCl_2_, 2 mM CaCl_2_, 10 mM D-glucose, and 10 mM HEPES (adjusted to pH 7.3 with NaOH). The solution was maintained at 37°C and bubbled gently with air. After stabilization, 10 μM forskolin and 50 μM genistein) were added to the both chambers, followed by the CFTR channel inhibitor [29] CFTRinh-172 (10 μM).

### Statistics

Western blots were evaluated by independent sample, T-Test and one-way ANOVA, followed by LSD post hoc tests. Statistical significance was set at P≤0.05, and data are presented as means±2SEM. All experiments were normalized to the respective controls and to Ezin. SPSS (version 17.0 SPSS, Inc, Chicago, III) was used for data analysis

## Results

### Characterization of N1303K and S1235 mutations

**Fig 1:**
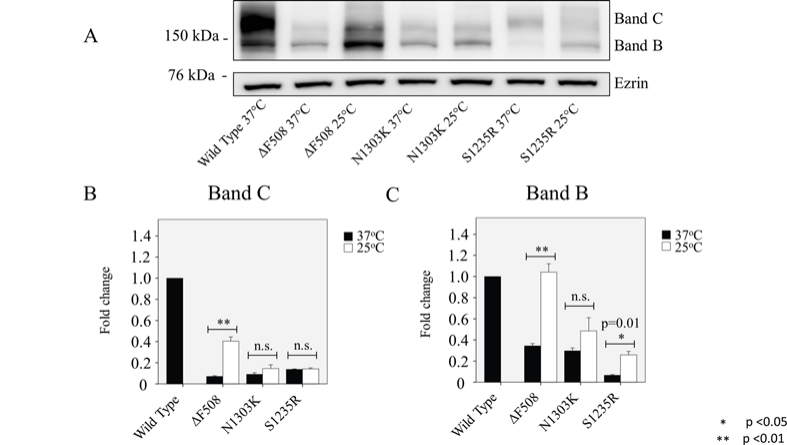
Characterization and temperature correction of N1303K and S1235R. **(A)** Cos7 cells were transfected with wild type (wt) CFTR, ΔF508, N1303K, or S1235R and then grown either continuously at 37°C or at 25°C for 24 h. Data are summarized in (**B and C).** Growing cells at reduced temperature significantly increases both the B and C bands of ΔF508, indicative of temperature rescue. Neither the B nor the C band of N1303K is increased by 25°C treatment. For S1235R, some increase in band B is seen, but no significant effect on band C. Thus, neither NBD2 mutant studied here responds to low temperature. Data are expressed as the mean ± SD of 3 independent experiments.

cDNA of N1303K and S1235R, wild-type CFTR, and ΔF508 was transfected into the Cos-7 cell line, and the protein expression of both mutations, N1303K and S1235R, was compared with wt CFTR and ΔF508, the most common CF mutation. Densitometry analysis showed that N1303K expressed a level of immature band B similar to that of ΔF508 and a barely detectable mature band C. S1235R expressed both band B and C again at lower levels compared to wt CFTR (Fig. 1). It is well known that growing cells at a reduced temperature (referred to as temperature rescue) can rescue the maturation of ΔF508-CFTR [5]. This is indeed what we observed for ΔF508-CFTR: an increase in both the immature band B and mature band C when we grew the cells at 24°C rather than 37°C (Fig. 1). In sharp contrast, the results differed for the NBD2 mutants: Temperature rescue had no effect on the maturation of band C of either N1303K or S1235R (Fig. 1). The only significant effect was an increase in the mature band B of S1235R when the cells were grown at the reduced temperature.

**Fig 2:**
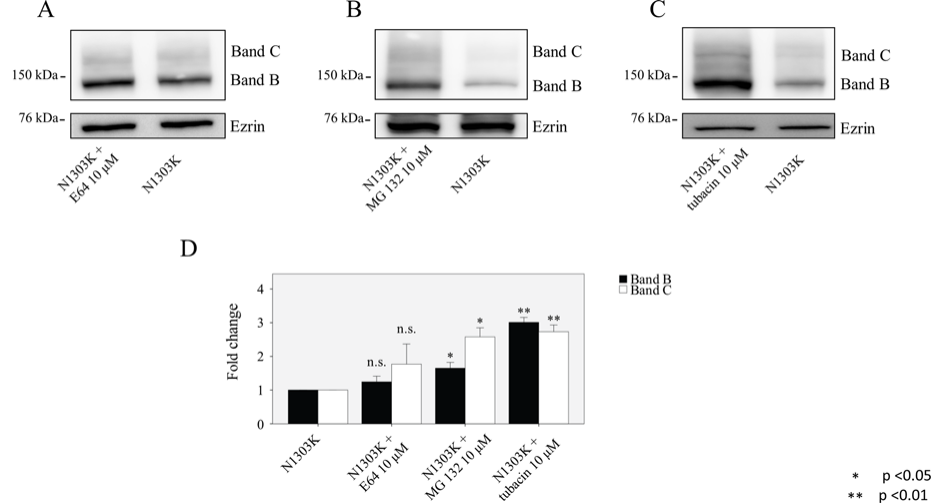
Degradation pathways for the N1303K mutation. An HEK 293 cell line stably expressing N1303K was treated with (**A)** the lysosome inhibitor E64, **(B)** proteasome inhibitor MG132, or **(C)** aggresome inhibitor tubacin. **(D)** The graph shows that the N1303K mutant is mainly degraded by the proteasome and aggresome; no effect was detected after lysosome treatment. Data are expressed as the mean ± SD of 3 independent experiments.

**Fig 3:**
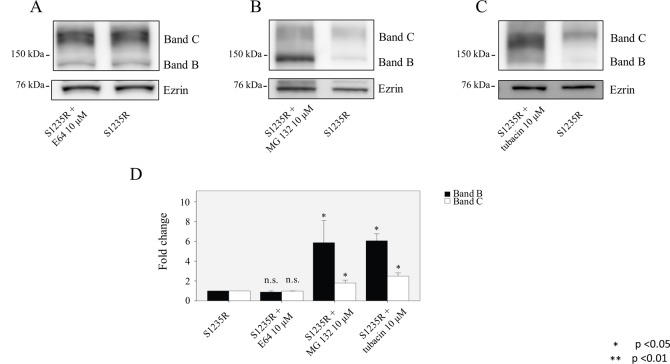
Degradation pathways for the S1235R mutation. An HEK 293 cell line stably expressing S1235R was treated with (**A)** the lysosome inhibitor E64, (**B)** proteasome inhibitor MG132, or (**C)** aggresome inhibitor tubacin. (**D)** The graph shows that the S1235R mutant is mainly degraded by proteasomes and aggresomes; no effect was detected after lysosome treatment. Cells were cultured for 16 hours at 37°C with or without the specified inhibitor (E64, MG132, tubacin. Data are expressed as the mean ± SD of 3 independent experiments.

Next, we applied inhibitors of proteasomes, aggresomes, and lysosomes in order to investigate the degradation pathway of the N1303K and S1235R mutants (Figs. 2 and 3). When we applied MG132, a non-specific inhibitor of proteasome degradation [12], the expression of band B and C in N1303K CFTR was significantly increased, indicating that this mutation is degraded in the proteasome. The effect was even more dramatic when we applied tubacin, an inhibitor of HDAC6, to block transport to aggresomes [30]. On the other hand, when we applied E64, an inhibitor of cysteine proteinases [31], to cells expressing N1303K, we saw no significant change in either the B or C band, indicating that this mutant is not degraded in lysosomes (Fig. 2). We saw an even greater increase in both the immature B and mature C bands of N1303K when tubacin was applied, this strong effect, especially on the immature B band, indicates a major role for aggresome formation [32] in the degradation of this mutant.

Like N1303K, the S1235R mutation was not affected by E64 inhibition. Interestingly, when we applied MG132 to cells expressing the S1235R mutant, we saw a 6-fold increase in the immature B band (Fig. 3). A similar effect was noted when tubacin was applied. In the case of both inhibitors, there was only a modest effect on the mature C band. Our results with the inhibitors indicate that cells handle these mutants quite differently.

### Application of small-molecule correctors has a significant effect in rescuing the protein expression levels of N1303K and S1235R

**Fig 4:**
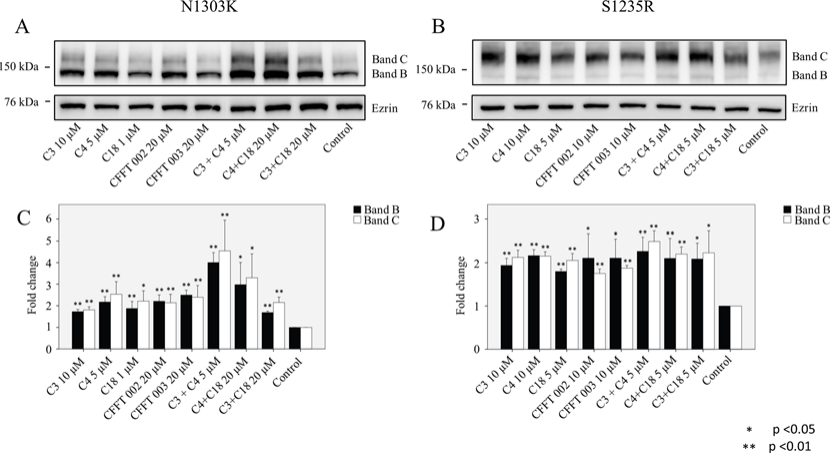
Rescue of N1303K and S1235R by small-molecule correctors. HEK 293 cell lines stably expressing either **(A)** N1303K or **(B)** S1235R were treated with compound alone (C3, C4, C18, CFFT 002, CFFT 003) or the combination of two compounds (C3+C4, C4+C18, or C3+C18). **(C and D)** Graphs show that all compounds are able to significantly increase bands B and C of N1303K and S1235R. The most significant effect occurred with combinations of C3+C4 and C4+C18. Cells were cultured for 16 hours at 37°C with or without correctors. Data are expressed as the mean ± SD of 3 independent experiments.

A number of corrector molecules have been shown to rescue ΔF508-CFTR [33], and the question we asked here is whether they would rescue mutants in NBD2. To address this question, we incubated cells with each of the following compounds for 16 h: C3, C4, C18, CFFT-002, and CFFT-003 (Fig. 4A, C). In the case of the N1303K mutation, most of the correctors increased both the B and C bands. However, the most significant effect occurred when combination of C3+C4 and C4+C18 was applied. Both combinations significantly increased the levels of B and C bands of N1303K, by at least two to three folds. In the case of S1235R, all correctors appeared to have a similar effect either alone or in combination, giving an increase of approximately two fold in both the B and C bands (Fig. 4B, D).

### Stability of both the B and C bands of N1303K and S1235R is significantly higher than that of the control, after treatment with corrector compounds

**Fig 5:**
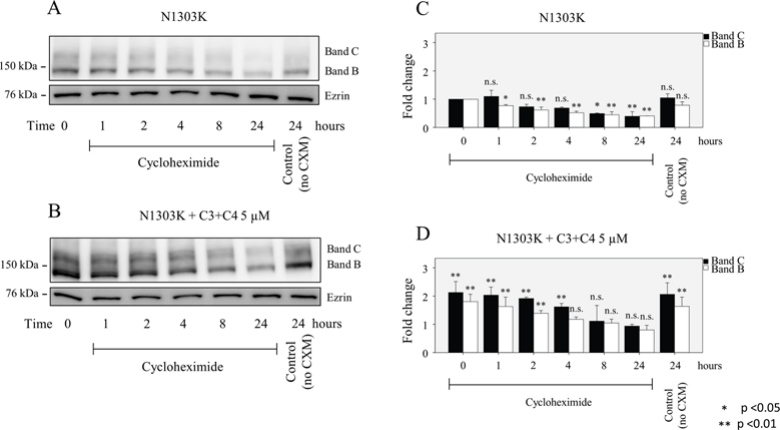
Half-live of N1303K with and without treatment with compounds. HEK 293 cell lines stably expressing either mutant were treated with cycloheximide (25μg/ml) and harvested after 1, 2, 4, 8, or 24 h. (**A, C)** The half-life of the C band of the N130K mutant is much shorter than the B band, which has a surprisingly long half-life. When C3+C4 5 (μM) was applied, the levels of both the B and C bands were higher at each time point for (**B, D)** N1303K. The major effect of the corrector combination was a prolongation of the half-life of the C band of N1303K. Cells were cultured for 16 hours at 37°C with or without correctors. Data are expressed as the mean ± SD of 3 independent experiments.

**Fig 6:**
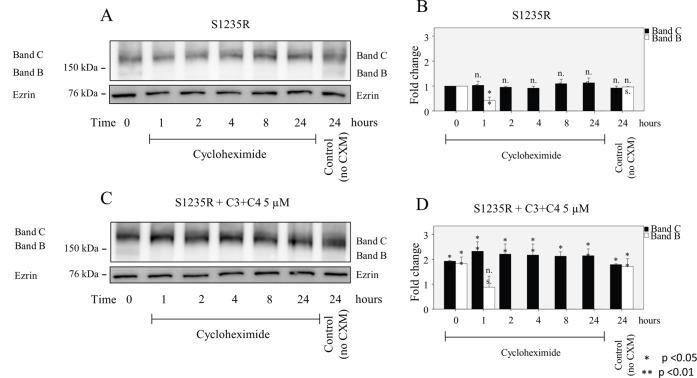
Half-live of S1235R with and without treatment with compounds. HEK 293 cell lines stably expressing either mutant were treated with cycloheximide (25μg/ml) and harvested after 1, 2, 4, 8, or 24 h. (**A, C)** The half-life of the C band of the S1235R mutant exceeds 24 h. When C3+C4 5 (μM) was applied, the levels of both the B and C bands were higher at each time point for S1235R **(B, D)**. Cells were cultured for 16 hours at 37°C with or without correctors. Data are expressed as the mean ± SD of 3 independent experiments.

Cells were treated with cycloheximide to inhibit protein synthesis [34] and then harvested at time 0 or after 1, 2, 4, 8, or 24 h. Western blot analysis showed that the half-life of the C band of N1303K was approximately 4–8 h (Fig. 5A, C). Interestingly, the B band of N1303K could be detected at 24 h. Similar measurements have shown that the mature C band of wt-CFTR is a relatively stable protein with a long half-life, whereas the half-life of the immature B band of ΔF508-CFTR is short, indicative of rapid degradation [35,36]. The mature C band of N1303K had a relatively short half-life when compared to that been reported for wt-CFTR, indicating that although some of the C band was processed, it was less stable than the mature C band of wt-CFTR. A similar observation has been made in the case of temperature rescue, which promotes the processing of an unstable mature band C of ΔF508-CFTR that is rapidly degraded when compared to the mature C band of wt-CFTR [36]. Unlike ΔF508-CFTR, whose immature B band has a very short half-life, the immature B band of N1303K was still detectable after 24 h. This stability may indicate that the processing of the N1303K mutant is arrested in the ER and is amenable to rescue by an appropriate combination of correctors such as C3+C4 or C4+C18 (Fig. 4). On the other hand, the half-life of the S1235R mutation exceeded 24 h (Fig. 6A, C), a half-life more closely resembling that of wt-CFTR [36]. When compounds C3 and C4 (at 5μM concentration) were applied to the mutants (5B,D & 6B,D) the levels of the B and C bands were higher at each time point; after 24 h, the cells treated with these two compounds exhibited levels of band B and C that were still higher than those of the corresponding untreated controls. Thus, it appears that corrector compounds exert their effect by increasing the amount of C and B bands in S1235R, whereas in the case of the N1303K mutant, the half-life is also increased.

### Small-molecule compounds increase the amount of mature CFTR localized to the plasma membrane

**Fig 7:**
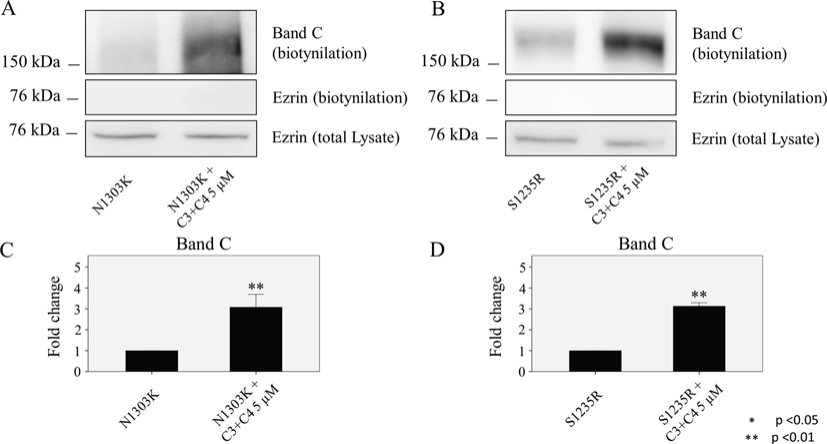
Effect of compounds on CFTR delivery to the plasma membrane. HEK 293 cell lines stably expressing either mutant cell line were treated with the combination of compounds having the best effect on the mutants. Compounds C3+C4 (5 μM) demonstrate the ability to significantly increase the delivery of the fully glycosylated protein to the plasma membrane, in the case of the N1303K (**A and C)** and S1235R (**B and D)** mutations. Cells were cultured for 16 hours at 37°C with or without correctors. Data are expressed as the mean ± SD of 3 independent experiments

Biotinylation was performed to determine whether the treatment with small-molecule correctors of N1303K and S1235R could increase the amount of band C in these CFTR mutants that was present at the plasma membrane. When compounds C3 + C4 at 5 μM were applied, they were able to increase by more than two fold the amount of mature CFTR capable of reaching the cell surface, as compared with the controls for both mutants (Fig. 7).

### Correctors reduce the binding of quality control proteins

**Fig 8:**
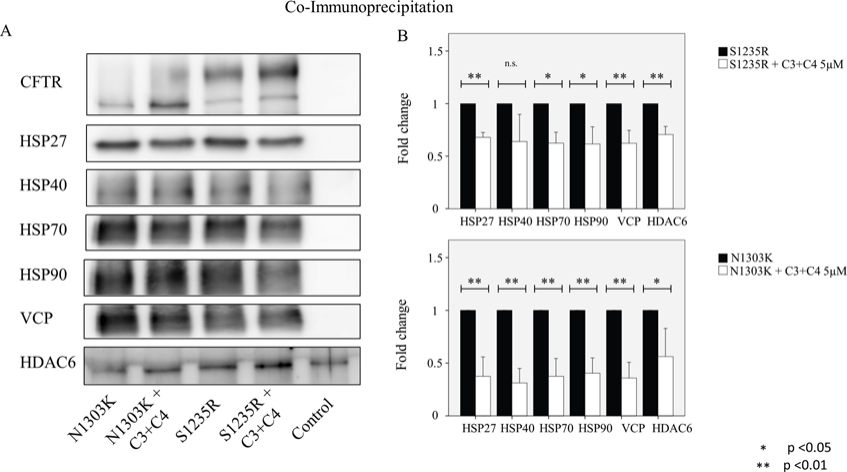
Co-immunoprecipitation of CFTR, HSPs, VCP, and HDAC6 in the N1303K and S1235R cell lines treated or untreated with the compounds. HEK 293 cell lines stably expressing either mutant were treated with C3+C4 (5μM), and CFTR was immunoprecipitated with anti-CFTR antibody M3A7. (**A)** Samples were used for western blot analysis and blotted with different antibodies as indicated in the figure. (**B)** Graphs were adjusted for the total amount of CFTR detected in the immunoprecipitated samples (the y axis shows the ratio between each sample and the total amount of CFTR in that sample. Controls are set as 100% binding). The data show that treatment with compounds C3+C4 at 5 μM is able to significantly decrease the amount of the HSPs VCP and HDAC6 that bind to the mutants. Cells were cultured for 16 hours at 37°C with or without correctors. Data are expressed as the mean ± SD of 3 independent experiments.

**Fig 9:**
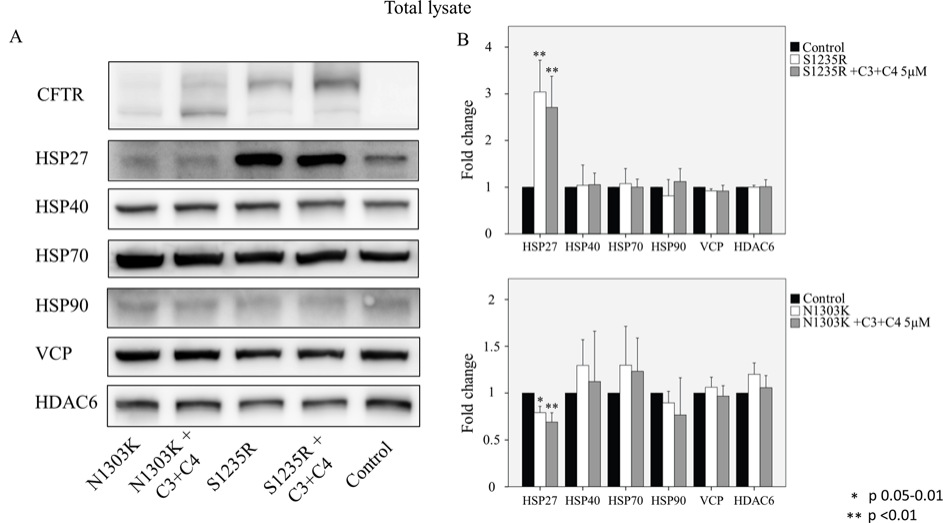
Total lysate levels of CFTR, HSPs, VCP and HDAC6 in the N1303K and S1235R cell lines. (**A)** Samples were used for western blot analysis and blotted with the various antibodies as specified in the figure. **(B)** The main difference in the total lysate is a significant increase in the total amount of HSP27 for S1235R and a significant but slightly decreased binding of HSP27 to N1303K when compared with the control (HEK 293 cell lines without any CFTR mutation). The graphs show that HSP27 in the cell line bearing the S1235R mutant, whether treated or untreated with the compounds, is four times higher than in the control and the N1303K cell line. No differences were found for all the other tested proteins (HSP40, 70, 90, VCP and HDAC6). No differences were found for HSPs, VCP and HDAC6 between the mutations alone or treated with compounds C3 + C4. Cells were cultured for 16 hours at 37°C with or without correctors. Data are expressed as the mean ± SD of 3 independent experiments

The interaction of ΔF508-CFTR with proteins involved with ER quality has been studied extensively, and many of the key players in this pathway are known to interact with misfolded CFTR [37]. Less is known about the interactions with mutants in NBD2. As shown in Figs. 8 and 9, a number of chaperones, including heat shock proteins (HSP) 27, 40, 70, and 90, bind to the N1303K and S1235R mutants. HSP70 and 90 are core ER chaperones that aid in the folding of wt-CFTR and the degradation of ΔF508-CFTR [38,39]. HSP40 is the co-chaperone for HsP70 [40]. The VCP protein is involved in translocation of mutant proteins to the proteasome [41]. Finally, HDAC6 is a protein involved in trafficking of mutant proteins to the aggresome [32]. It is significant that all the interactions involved in the degradative pathway, including the chaperones, VCP, and HDAC6, are reduced following treatment with the combination of correctors (Fig. 8), considerably strengthening our contention that the combination of C3+C4 rescues both mutants (Fig. 4). It is also noteworthy that the reduction in binding was greater with the N1303K mutant than with the S1235R mutant (Fig. 8B). Given that N1303K causes a more severe form of CF than does S1235R, these results may indicate a more avid engagement of N1303K with the degradative process. The binding data precisely paralleled the increase in the rescue of mature C band of N1303K, which was increased by more than 2 folds by the combined treatment with C3+C4, as compared with the rescue of S1235R (Fig. 8B).

Total lysate shows similar levels of HSPs, VCP and HDAC6 in both mutations alone or treated with compounds C3 + C4. The main difference in the total lysate is a significant increase in the total amount of HSP27 for S1235R and a significant but slightly decreased amount of HSP27 for N1303K when compared to the control (HEK 293 cell lines without any CFTR mutation) (Fig. 9).

### Transcomplementation by Δ27–264 is able to rescue NBD2 mutants

**Fig 10:**
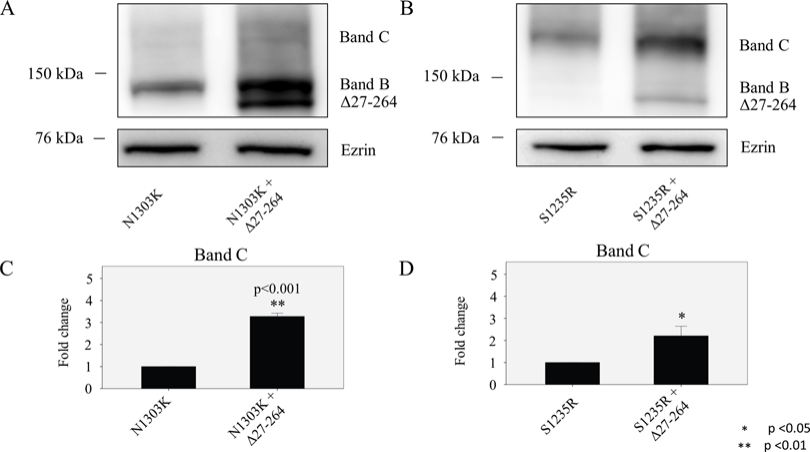
Effect of transcomplementation with Δ27–264 on N1303K and S1235R mutants. Δ27–264-CFTR was transfected into HEK 293 cell lines stably expressing the mutants. Δ27–264-is able to increase significantly band C of (**A)** N1303K and (**B)** S1235R. Graphs show that the fully glycosylated protein is increased by 3 times for (**C)** N1303K and more than 2 times for **(D)** S1235R. Cells were cultured for 48 hours at 37°C with or without the truncated construct (Δ27–264). Data are expressed as the mean ± SD of 3 independent experiments.

**Fig 11:**
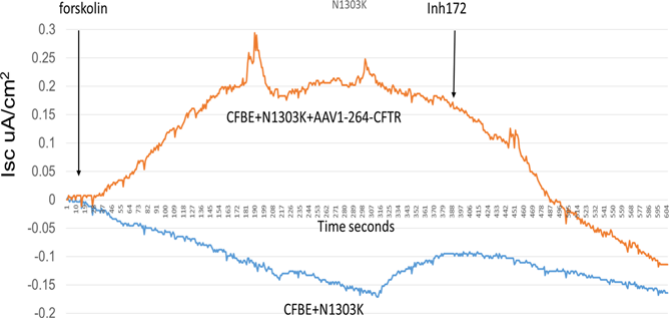
Transcomplementation rescues N1303K CFTR currents. CFBE cells stably expressing N1303K-CFTR were grown on Transwell supports and treated with either 10 μl of AAV1 containing Δ264 CFTR as described in Materials and Methods. In the graph, 10 μM forskolin was added along with genistein, and 10 μM CFTRinh-172 was added at the second arrow to demonstrate that the currents were from CFTR. Note the large increase in currents when compared to a cell not exposed to virus (representative of three experiments).

We have previously shown that truncated forms of CFTR such as Δ264 and Δ27–264-CFTR can rescue the NBD1 mutants ΔF508-CFTR and A455E CFTR [27,42], but it was still not clear whether the NBD2 mutants could also be transcomplemented. Transient transfection of the Δ27–264 plasmid into the N1303K and S1235R HEK Flp-In-293 stable cell lines was able to rescue the N1303K mutation, increasing the band C expression by 3-fold when compared to the control (Fig. 9A). Treatment with the CFTR truncated form Δ27–264 was also able to rescue band C of S1235R, increasing it by more than 2-fold (Fig. 9B). Importantly, when the chloride transport ability of the N1303K mutant was measured in CFBE stably expressing the mutant, we saw a dramatic increase in Isc in the cells infected with AAV1 containing Δ264-CFTR. We have shown previously that Δ264-CFTR by itself does not generate chloride channel currents, but it can rescue chloride currents in ΔF508-CFTR[27]. Thus, transcomplementation can rescue both the trafficking (Fig. 10A, B) and chloride transport of the N1303K mutant (Fig. 11).

## Discussion

Here we have studied two mutations in NBD2, S1235R and N1303K, and showed that both can be rescued by small-molecule correctors, used in combination, and transcomplementation using a truncated version of CFTR. The N1303K mutation is associated with severe CF and is a processing mutant in NBD2, with minimal chloride conductance and mature C band at the cell surface when assessed in rat Fischer thyroid cells (FRT) and HeLa cells [43]. The asparagine resides at 1303, located within the alpha-helical subdomain, is also located in a similar position in NBD1 at residue 505, three resides from the missing phenylalanine at amino acid 508. Interestingly, the asparagine is also highly conserved among members of the ABC transporter family, including pgp1, mrp1, HisP, and TAP1 [44]. Given the role of asparagine in hydrogen bonding, it is likely to play a critical role in stabilizing NBD2 in wild-type CFTR and in interactions between NBD2 and the transmembrane domains. The lysine residue, on the other hand, is disruptive, as suggested by the poor function and trafficking of N1303K; it could also potentially serve as a site for abnormal ubiquitination, further disrupting the normal trafficking of CFTR. S1235R, in contrast, is associated with mild or minimal disease [43]. The serine at this position, although present in both human and mouse, is not highly conserved [44]. In NBD1, the equivalent amino acid is glutamic acid, and thus the arginine replacement in S1235R seems to be mildly disruptive; the conductance measured in FRT cells is approximately 79% of wt-CFTR, and the protein processing is nearly equivalent to that measured for the G551D mutant [43]. Clearly, these mutations located in different parts of NBD2 would be expected to have different structural and functional effects on the final assembly of CFTR.

What is striking about the NBD2 mutants is that they behave differently in response to temperature rescue. Unlike ΔF508-CFTR and A455E, both of which undergo significant trafficking of immature band B to mature band C [12], the mature bands of N1303K and S1235R are not enhanced when cells are grown at low temperature. This lack of temperature rescue in the NBD2 mutants may be indicative of the difference in the way each domain achieves its final structural conformation. Whereas NBD1 is folded as CFTR is translated [15], there is some evidence that NBD2 is folded post-translationally as a final step in allowing CFTR to achieve its mature conformation [45]. Other evidence suggests that all the domains of CFTR are folded during translation [15]. However CFTR folds, it is clear that these mutations in NBD2 do not achieve a mature conformation. Both mutations are sensitive to proteasomal and aggresomal inhibitors, suggesting that they are degraded by processes involved in the degradation of membrane proteins. S1235R is particularly sensitive to tubacin, the aggresome inhibitor. This response of S1235R is in sharp contrast to that of A455E, which is not sensitive to tubacin. There was surprisingly little effect of lysosomal inhibition, especially on the S1235R mutant, even though this mutant does have significant quantities of mature C band at the plasma membrane. When protein translation is inhibited by cycloheximide, the immature B band of N1303K is more stable than ΔF508-CFTR, which is rapidly degraded and disappears rather quickly (see [42]). On the other hand, the small amount of mature C band that is processed is short-lived, indicative of an unstable mature band. These results point to differences in the processing of these NBD2 mutants when compared to two of the mutations in NBD1, A455E and ΔF508-CFTR.

The question, then, is that given these differences, can NBD2 mutants be rescued with correctors designed to rescue NBD1? To study the correction of the NBD2 mutant by small molecules, we tested the effect of numerous compounds: C3, C4, C18, CFFT 002, and CFFT 003; C3, C4, and C18. The latter three were also used in combination. Bands B and C of N1303K were significantly increased when small-molecule correctors were applied, with an additive effect when they were administered together. Similar results were found for S1235R; all compounds were effective to some extent when administered individually. However, when they were administered together, we saw a less remarkable effect than we had observed for N1303K. In order to investigate whether this effect was related only to the CFTR steady-state or instead to increased maturation of the chloride channel, we performed biotinylation experiments with the combination of compounds that showed the best effect (C3+C4 at 5μM). The results showed a significant increase in the presence of the fully glycosylated bands of both N1303K and S1235R at the cell surface when C3+C4 was applied. Thus, combined corrector therapy was able to increase their CFTR maturation and the delivery to the plasma membrane.

Recently, the correctors we utilized here have been divided into different classes: C3 and C18 were assigned to Class I, the correctors that stabilize the NBD1- CL1–4 interface. C4 was assigned to Class II, those correctors that restore NBD2 or its interfaces. The correctors CFFT 002 and 003 were not included in the classification scheme [33]. Interestingly, although C4 is purported to affect NBD2 or its interface, in our hands it had an effect on both NBD2 mutants, but the effect was not as striking as that of the others we tried. The most profound effect was noted for N1303K, when we combined a Class I corrector with one from Class II, indicating that mutations in NBD2 may also have effects on the other parts of CFTR, which themselves will have to be corrected. Interesting, the combination of C3+C4 had a large impact on the interaction of N1303K with proteins associated with ERAD, another indication of their combined effectiveness.

In the past few years, our group has demonstrated that transcomplementation of ΔF508 can rescue both its processing and function. In particular, we have shown that Δ264, a CFTR lacking the first 264 amino acids, is able to increase the levels of immature band B in ΔF508, leading to augmentation of its processing to mature band C. The main mechanism of action in this case is interference with the VCP-HDAC6/CFTR complex, promoting the translocation of mature ΔF508 to the cell surface [42]. Later, this construct was modified by adding the first 27 amino acids of wt-CFTR to the N-terminus of the Δ264 construct (Δ27–264). This improvement allowed Δ27–264-CFTR to take advantage of the same mechanism as Δ264, but also promoted the direct binding of the new construct to ΔF508, thereby increasing the amount of the mature protein present at the plasma membrane and rescuing the chloride channel gating function[27]. We show here that transfection of the Δ27–264 plasmid does indeed lead to rescue of the fully glycosylated band of N1303K and S1235R. Interestingly, the effect of Δ27–264 was comparable to that of most of the chemical correctors tested. Because Δ27–264 is able interact with ΔF508 through a bimolecular interaction, we believe that a similar mechanism may also rescue the NBD2 mutants, allowing Δ27–264 CFTR to act as a molecular chaperone to help the NBD2 mutants to achieve a more native state [27,42]. Alternately, given the effect of Δ264 CFTR on key players in the ERAD pathway, it is possible that transcomplementation of the NBD2 mutants occurs through a disruption of their processing by ERAD, allowing more C-band to reach the plasma membrane.

### In summary

Our results suggest that the N1303K mutation has a more profound effect on CFTR processing than does S1235R, consistent with the observation that N1303K causes severe CF and S1235R only mild-to-borderline disease [19,23,46]. We show here that these two NBD2 mutants of CFTR can be rescued by small-molecule correctors that were shown to be effective not only in increasing the levels of B and C bands in the NBD2 mutants but also in dramatically increasing the delivery of mature protein to the plasma membrane. Both NBD2 mutants are capable of transcomplementation with truncated forms of CFTR. Finally, our results suggest that patients with N1303K or S1235R mutations may be good candidates for therapy with small-molecule correctors, combined with transcomplementation with the truncated form of CFTR, Δ27–264.
